# Defending the city’s cleanliness with their lives? A study of road traffic collisions involving sanitation workers in China over five years

**DOI:** 10.1186/s12889-021-11977-1

**Published:** 2021-11-02

**Authors:** Lifeng Wei, Zhuowa Sha, Haonan Jia, Yidong Wang, Gangyu Zhang, Yuanheng Li, Yameng Wang, Shuang Zhou, Ying Wang, Chao Liu, Mingli Jiao, Jingfu Mao, Qunhong Wu

**Affiliations:** 1 Harbin Medical University, Nangang District, 157 Baojian Road, Nangang District, Harbin, 150086 Heilongjiang China; 2 Heilongjiang Provincial Health Development Research Center, Xiangfang District, Harbin, Heilongjiang China

**Keywords:** Road traffic collisions, Sanitation workers, Urban governance, Vulnerable groups

## Abstract

**Background:**

With increasing urbanization in developing countries, sanitation workers are frequently involved in road traffic collisions. Our purpose was to study specific collisions involving sanitation workers and provide decision-making suggestions and reference measures for the sanitation industry and urban managers to reduce the occurrence of collisions.

**Methods:**

We obtained online news data about sanitation worker road traffic collisions in China between 2013 and 2017 and analyzed occurrence time and location, victim characteristics, and causes of collisions.

**Results:**

In China, between 2013 and 2017, 511 road traffic collisions were reported, with the fewest in February and July. Most occurred around 5:00 a.m. in Eastern regions and in urban areas. Victims were mainly over 50 years old, with more females than males. Collisions usually resulted in death at the scene. The ambiguity of laws, the exploitation of workers through industry outsourcing, and the difficulty of processing claims may be the main factors preventing victims from obtaining legal compensation.

**Conclusions:**

The most common cause of collisions was drivers’ speeding, but workers also regularly risk death by crossing the road in pursuit of their duties. The absence of legal controls for environmental protection, the excessive pursuit of efficiency in urban governance, and the lack of basic education of sanitation workers are underlying causes of collisions. Raising awareness about sanitation worker road traffic collisions will help protect the work safety rights of this vulnerable group.

## Background

Over the past few years, Chinese sanitation workers have encountered hundreds of road traffic collisions, resulting in many victims. According to our ongoing statistical research, about 10% of sanitation worker road traffic collisions analyzed in this study report multiple victims.

Sanitation workers (garbage collectors or solid waste collection workers) are an occupational group that deals with sanitation, and their road traffic collisions are closely related to urbanization. Worldwide, urbanization shows no sign of slowing down, especially in developing countries [1–3]. On the one hand, urbanization brings about the expansion of urban populations and a corresponding increase in car ownership and road traffic congestion, which increases the possibility of road traffic collisions [4–6]. Among the top 10 causes of death in the world in 2016, road traffic injuries ranked eighth [7]. Nearly three-quarters of global road traffic deaths occurred in developing countries, with China accounting for 21% as the most populous country in the world [8, 9]. On the other hand, with the expansion of urban populations, environmental and sanitation problems arise, such as the exponential growth of urban garbage [10]. The demand for urban cleanliness increases proportionally with the level of urbanization. In contrast to the highly mechanized cleaning methods in developed countries, abundant, low-cost labor resources in developing countries mean that cleaning is mainly done by people rather than machines, resulting in a large number of sanitation workers who play an indispensable municipal function [11, 12].

However, Chinese sanitation workers differ from those in other developing countries in working mainly in public places dominated by urban roads and undertaking part of the garbage transportation from public waste containers (e.g., waste bins on both sides of the road) to waste transfer stations. The workers do not perform subsequent garbage sorting and processing [13]. The fact that sanitation workers work mainly on the road exposes them to more traffic collisions. Further, in China it is common to see fallen debris from waste removal, litter, and objects thrown from buildings into the street. People in vehicles on the road are free to throw waste out of windows (colloquially referred to as the “window littering”), and 90% of waste on the road is from private cars, taxis, and buses [14]. In order to deal with this waste, Chinese sanitation workers are at high risk of experiencing collisions as they are frequently required to cross roads in the course of their duties. Workers are often placed in the position where they are required to risk their own lives in order to maintain the cleanliness of cities.

Excessive road traffic injuries and high collision risks make sanitation workers a vulnerable urban group. If the current situation does not change, the occurrence of road traffic collisions involving sanitation workers may increase with population growth in urbanizing countries with low levels of mechanization. Cointreau listed vehicle collisions as one of the occupational health risk factors for solid waste collection workers [15]. A study of urban sanitation workers in South Korea showed that 20.3% of sanitation worker injures were due to traffic collisions during road cleaning [16]. An Indian study showed that the occupational injury rate of solid waste collection workers is higher than that of general workers; road traffic collisions account for 22% of injures [17]. A study of occupational injuries among Brazilian garbage collectors also found collisions between vehicles and workers [18].

Although road traffic collisions are a risk factor in the occupational health and safety of sanitation workers, no previous research has specifically analyzed the conditions for and underlying causes of collisions or interventions to curb them. Research in China, the largest developing country, is particularly lacking, especially considering the high incidence of such traffic collisions. Relevant online news articles are, however, abundant and provide sufficient research material. The purpose of this study is to 1) study relevant online news on specific road traffic collisions involving Chinese sanitation workers; 2) analyze data on occurrence time and location, characteristics of victims, and causes of collisions; and 3) provide actionable suggestions and implementable measures for the sanitation industry and urban managers to reduce the occurrence of sanitation worker road traffic collisions.

## Methods

**Fig. 1:**
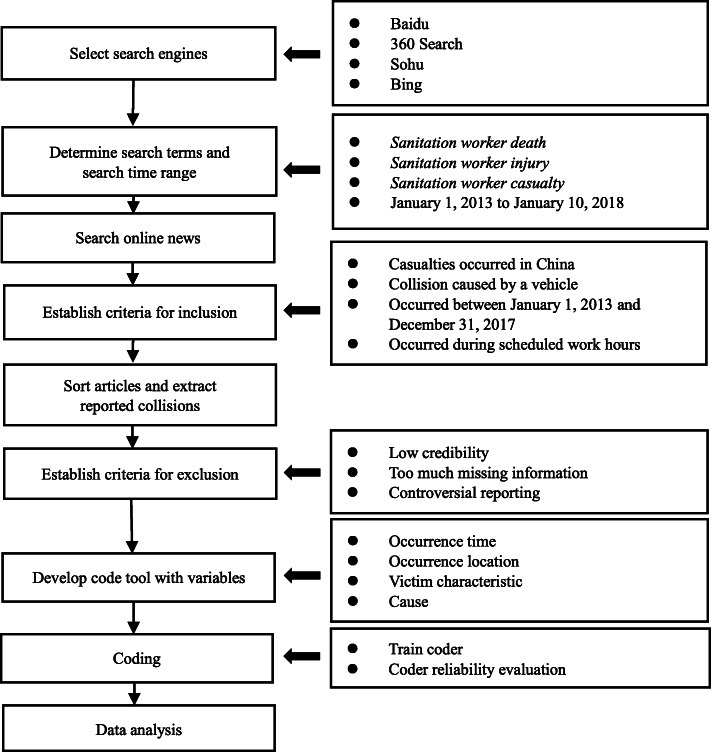
Flow chart of research method

Drawing from the methods used to investigate online news about violence against doctors in China and then quantify the characteristics of violence [19], this study examined online news about road traffic collisions involving sanitation workers (Fig. 1). By searching, screening, categorizing, and encoding these relevant online news, occurrence time and location, victim characteristics, and causes of the reported collisions were obtained and analyzed.

### Sample

Over 3 weeks in May 2018, we searched online news stories for sanitation worker road traffic collisions, choosing four major Chinese search engines: Baidu, 360 Search, Sohu, and Bing. Three productive Chinese search terms were *sanitation worker + death, + injury*, and *+ casualty*. Due to the delay of online news reporting, the reporting period examined was from January 1, 2013 to January 10, 2018.

Injuries to sanitation workers not caused by vehicular impact or outside the context of work were beyond the scope of our study. Our inclusion criteria narrowed our focus to incidents in China between January 1, 2013 and December 31, 2017 involving injury caused by both motor and non-motor vehicles to sanitation workers during their work hours.

Hundreds of thousands of web hits were generated, not all unique news stories. To narrow our results, we stopped search engine inclusion after 500 consecutive news without a new collision. Then we sorted the articles according to reported collisions and extracted them, excluding collisions with low credibility (unknown news source, wrong wording and language, etc.), excessive information loss (complete absence of information on time, location, casualty, and cause), and controversial reporting (information from news reports on the same collision was divided). We reviewed and coded 511 sanitation worker road traffic collisions.

### Measurement

To code the collisions, we developed a comprehensive table containing specific variables about collision occurrence time and location, victim characteristics, and cause of collisions. All collisions were systematically coded by a team of four well-trained coders. We used Cohen’s kappa to evaluate the reliability between the coders by having them code 25% of the samples separately. The reliability scores of this study ranged from 0.71 (lowest) to 1.0 (highest), indicating a high degree of agreement between the four coders. The remaining 75% of the collisions were then coded.

### Data analysis

The frequency distribution calculation was performed on the data recorded on the coding table. Analysis was performed using SPSS V.20.0 (IBM). We adopted a strategy to analyze only valid collisions, as shown in Table 1.

**Table 1:** Number of valid collisions for variables

	Number of valid collisions (*N* = 511) n(%)
**Occurrence time**	
Date of collision	511 (100.00)
Time of collision	466 (91.19)
**Occurrence location**	
Province where collision occurred	511 (100.00)
Area where collision occurred (Urban, Rural)	511 (100.00)
Region where collision occurred (East, Central, West)	511 (100.00)
**Victim characteristic**	
Number of victims	509 (99.61)
Sex of victims	352 (68.88)
Age of victims	320 (62.62)
Severity of injury (Injury, Death)	501 (98.04)
Place where the deceased died (Died on the scene, Died en route to the hospital, Died in hospital)	322 (63.01)
Sources of victim compensation (Compensation by driver, Company subsidy, Personal accident insurance payment, Government subsidy, Employment injury insurance payment)	89 (17.42)
**Cause**	
Types of cause	304 (59.49)

## Results

**Fig. 2:**
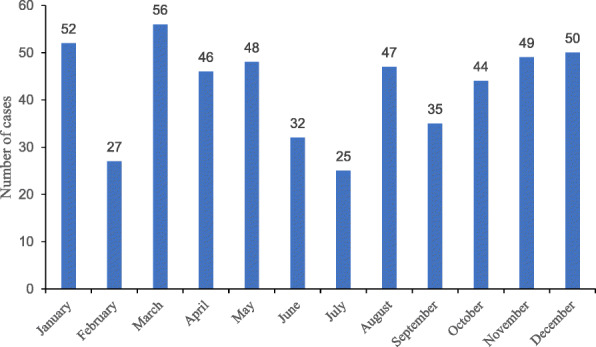
Occurrence of sanitation worker road traffic collisions by month (*n* = 511)

Most months, between 32 and 56 collisions occurred (Fig. 2), but each February and July there were fewer collisions.

**Fig. 3:**
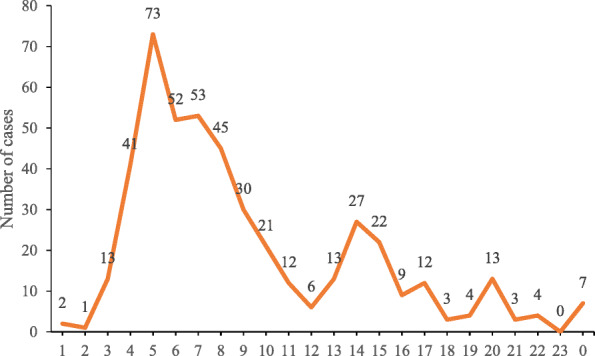
Occurrence of sanitation worker road traffic collisions by hour (*n* = 466)

A majority (63.1%) of collisions occurred between 4:00 and 9:00 a.m., and 15.7% of collisions occurred at 5:00 a.m. (Fig. 3). The number of collisions peaked again slightly at 14:00, 20:00, and 0:00.

**Fig. 4:**
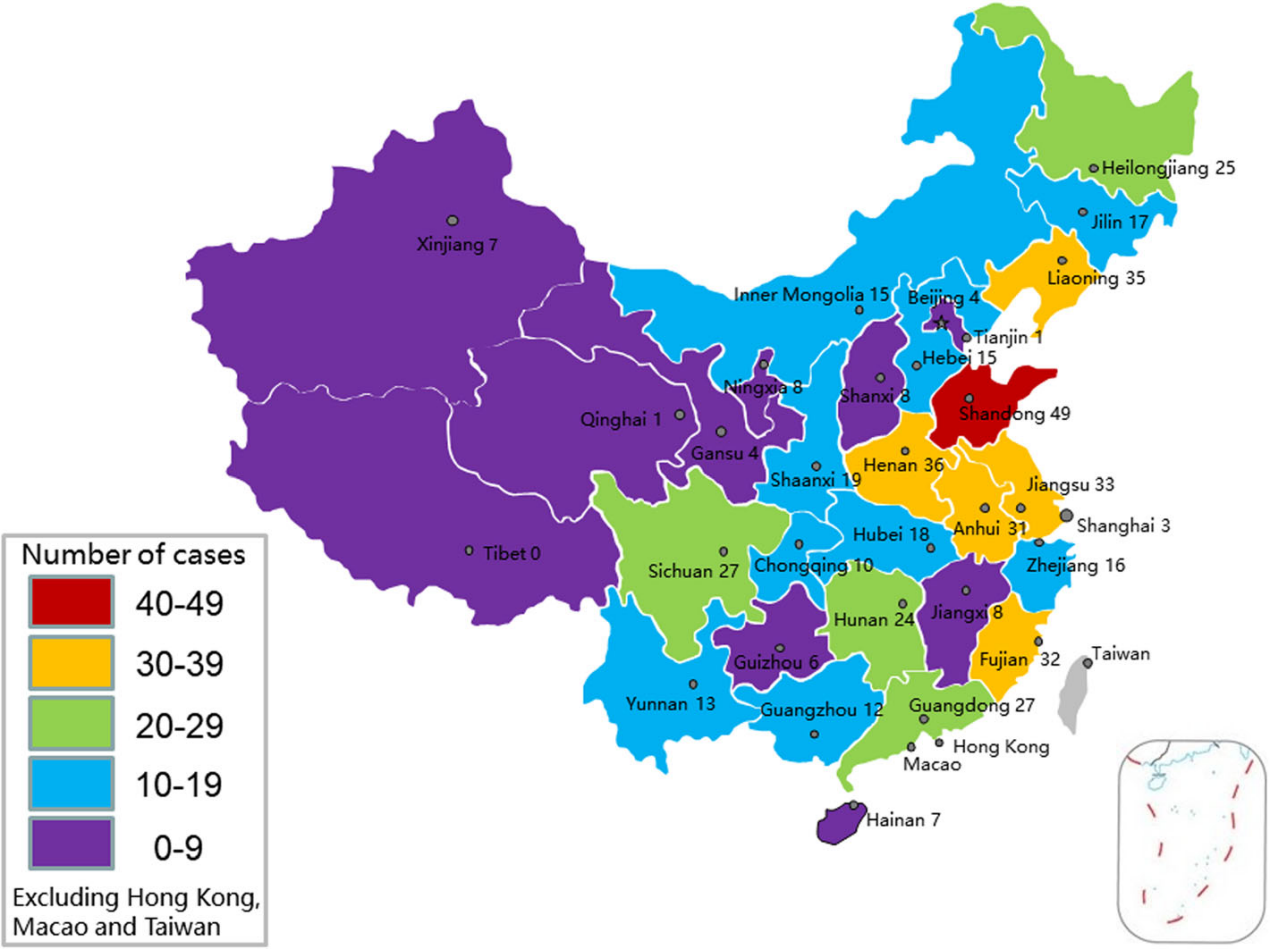
Occurrence of sanitation worker road traffic collisions by province in China (*n* = 511). Note: The map depicted in the figure was produced by Microsoft Office PowerPoint

This study determined the number of sanitation worker road traffic collisions in various provinces of China, excluding Hong Kong, Macao, and Taiwan (Fig. 4).

Table 2 summarizes the distribution of collisions by area and region types. The number of urban collisions was far higher than rural collisions. All Chinese provinces are divided into eastern, central, and western regions according to economic and technological development level and geographical location. Collisions occur mostly in the eastern region, followed by the central region and the western region.

**Table 2:** Occurrence of sanitation worker road traffic collisions by location

	N	%
**Area type**	511	
Urban	496	97.1
Rural	15	2.9
**Region type**	511	
East	223	43.6
Central	166	32.5
West	122	23.9

Out of the 509 collision reports that mentioned a specific number of victims, there was a single victim in 91.0% of cases and multiple victims 9.0% of the time. Thirty collisions had two victims each, eight collisions had three victims, and other collisions had up to seven victims each. Sanitation workers died in 326 collisions (64.0% of the total).

Records of sex and age were available for 383 and 342 victims respectively (Table 3). The number of female victims was about 1.5 times that of male victims. Most of the victims (76%) were 50 and older, while the number of victims aged 30–39 was the lowest (2.9%). Collisions resulted in injuries and, most often, deaths at the scene (Table 3).

**Table 3:** Characteristics of sanitation worker traffic collision victims

	N	%
**Sex of victim**	383	
Male	149	38.9
Female	234	61.1
**Age of victim**	342	
30–39	10	2.9
40–49	72	21.1
50–59	127	37.1
≥ 60	133	38.9
**Severity of injury**	575	
Injury	227	39.5
Death	348	60.5
**Place of death**	344	
Died on the scene	229	66.5
Died on the way to the hospital	5	1.5
Died in hospital	110	32.0

**Fig. 5:**
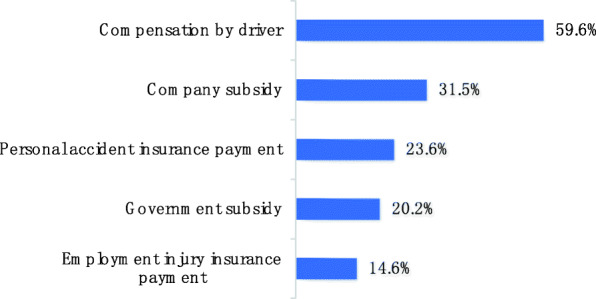
Source of compensation of sanitation worker traffic collision victims (*n* = 89)

For each collision, there could be multiple sources of compensation for victims (Fig. 5). Most frequent was compensation by driver and least frequent was employment injury insurance payment.

**Fig. 6:**
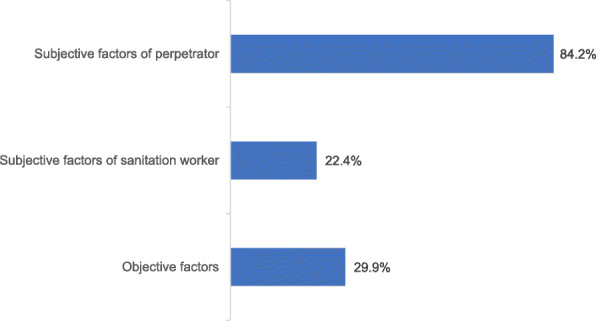
Reported cause for sanitation worker road traffic collisions by aspect (*n* = 304)

We divided reported causes of collisions into subjective factors pertaining to the perpetrator or the sanitation workers, and objective factors such as bad weather and dim light (Fig. 6). The subjective factors of the perpetrator occurred the most frequently, while the frequency of subjective factors of the sanitation worker and objective factors were essentially the same.

For perpetrator-caused collisions, the top three factors were speeding, negligence, and drunk driving. Speeding was by far the most frequent cause of collisions, and also the most dangerous for the perpetrators themselves. The negligence of perpetrators in news reports specifically included dangerous behaviors such as talking on the cell phone while driving and not paying attention to the front. For collisions caused by sanitation workers, crossing the road at will was the most common factor. Violation of traffic rules, as well as illegal operations such as backing on traffic, not wearing reflective clothing, and not setting up warning signs or officers, were also dangerous factors from the sanitation workers themselves. Bad weather (e.g., rain, snow, and fog) and dim light were the two objective factors with the highest occurrence frequency, although other factors appeared occasionally (Table 4).

**Table 4:** Specific reported cause for sanitation worker road traffic collisions

	N	%
**Subjective factors of perpetrator**	256	
Speeding	137	53.5
Negligence	67	26.2
Drunk driving	28	10.9
Unlicensed driving	19	7.4
Sudden lane change	17	6.6
Fatigue driving	14	5.5
Reverse driving	9	3.5
Running a red light	2	0.8
**Subjective factors of sanitation worker**	68	
Crossing the road at will	35	51.5
Violation of traffic rules	21	30.9
Illegal operation	21	30.9
**Objective factors**	91	
Bad weather (rain, snow, fog, etc.)	24	26.4
Dim light	21	23.1
Traffic disorder	16	17.6
Obstruction of sight of the road line	11	12.1
Vehicle failure	10	11.0
Debris on the road	7	7.7
Problems with road warning signs or traffic lights	6	6.6
Dazzling light	5	5.5

## Discussion

The present research aimed to investigate the characteristics of road traffic collisions involving Chinese sanitation workers. We summarized occurrence time and location, characteristics of the victims, and causes of the collisions with the goal of providing decision-making suggestions and reference measures to reduce the occurrence of sanitation worker road traffic collisions.

Our research revealed that 5:00 a.m. is the peak time for collisions, probably because there is generally less traffic on the road at this early hour, which gives general road vehicles the opportunity to speed. In most parts of China, at 5:00 a.m., the sky is likely to be dark and foggy, which makes for poor visibility and increases the risk of collisions [20]. Furthermore, most Chinese sanitation workers start working in the early morning to complete a thorough road cleaning before traffic peaks [11, 21], which may be related to the spike in collisions around 5:00 a.m.

There are almost no sanitation workers in the vast rural areas of China. Most sanitation worker road traffic collisions occur in urban areas, a finding in line with past research about traffic injuries in developing countries [22]. A 2014 dataset shows that the urbanization rate of permanent residents in the eastern region of China reached 62.2%, while that in the central and western region was only 48.5 and 44.8% respectively [23]. High urbanization is often accompanied by high levels of economic development, and regions and provinces with high levels of economic development have more sanitation worker road traffic collisions.

A study on sanitation workers in Wuhan, Hubei Province, found more female than male sanitation workers [11], and our study found that women were the main collision victims. Therefore, female sanitation workers may need more support and occupational protections. However, due to the unavailability of personal information of China’s overall sanitation workers, we cannot yet determine whether this higher frequency of female victims was proportionate to the number of female sanitation workers. Whether female sanitation workers have a higher risk of road traffic collisions also needs to be further explored. Like sex, age is a salient factor; studies about sanitation workers in China concluded that most are elderly [11, 24], and in our results, most collision victims were 50 and older. The social status of sanitation workers is generally low, but in developing countries it is even worse [25]. The stigma associated with touching trash, low wages for sanitation workers, and the higher age of most sanitation workers discourage young people from doing such work [24]. Moreover, an occupation with low entry barriers (no education or technology requirements) may be an easier choice for the elderly. Finally, older people’s response time and agility is lower compared to younger people, which makes it more difficult for older people to avoid impending road traffic collisions. Therefore, mandatory medical fitness (initial and periodical) by the employers through legislation may play a certain role in ensuring that elderly sanitation workers are qualified for work.

Among the victims of sanitation workers, there were far more deaths than injuries. This may indicate that road traffic collisions involving sanitation workers often caused serious consequences. However, Bias of data collection from online news that tends to report serious collisions may also be an obvious reason.

Since perpetrators generally bear part of the responsibility in most road traffic collisions, it is reasonable for compensation by driver to be the most common source of victim compensation (60%). The collisions we studied all occurred during working hours, but employment injury insurance was the least common form of payment. Although we have no convincing data to support, we do have some inferences about the causes of this phenomenon based on 89(17.42%) valid collisions, which need to be confirmed by more research.

First, legal provisions are ambiguous. In China, employment injury insurance is entirely paid by the employer. Many laws clearly stipulate the obligation of employers to pay for insurance and the right of employees to obtain work-related injury compensation, but for employees who reach the statutory retirement age (60 years for men, 55 for women), eligibility to participate in insurance plans is controversial, and the qualifications for work-related injury identification are different in various provinces and cities. The aging of sanitation workers and the fact that most victims are 50 or older make these problems more apparent. Vague legal provisions may be an excuse for employers not to purchase employment injury insurance for sanitation workers.

Second, there is a strong possibility that outsourcing contractors exploit sanitation workers. China’s sanitation services began to move from the public sector to the private sector in the 1990s [21, 26]. The outsourcing of Chinese sanitation services may have the advantage of cost savings and efficiency gains, and there is no reason to believe that profit-driven private companies will reduce service quality [27, 28]. Cost, however, is transferred to sanitation workers through a decline in wages, job security, and welfare, the degradation of working conditions, and increased workload [21]. Driven by profit, employers may choose more profitable methods that undermine accountability and even violate human rights, such as restricting salaries, canceling benefits, and unreasonably increasing workloads; not purchasing employment injury insurance for sanitation workers is a simple and practicable way for employers to save money. As Aguiar described in his study of Canada, cleaning work is becoming “sweatshop work” [29]. The Chinese government has facilitated for-profit outsourcing and fails to take into account the rights of employees in contract development and operations, focusing only on whether cleanliness is up to standard and not on how those standards are attained. Outsourcing sanitation service does not mean outsourcing government responsibility, and the Chinese government has not yet lived up to its responsibility to sanitation workers.

Third, claiming compensation through employment injury insurance is difficult. The claimant is required to go back and forth between the employer, the traffic police, medical institutions, labor security administrative departments, and other related institutions, and provide more than ten kinds of documentation. Moreover, any disputes arising in the process need to be resolved through litigation, which costs time, money and effort. Thus, many people may choose to waive their claims.

In our study, we found that 23.6% of victims needed to pay for medical expenses or maintenance expenses themselves due to lack of compensation. When sanitation workers do not have sufficient wages or employment injury insurance and the drivers do not pay, it is crucial to establish an external multi-party assistance mechanism including government finance, commercial insurance, and social assistance [15].

Drivers do not pay compensation in 40% of cases, although excessive speed was identified as one of the most common contributors to traffic collisions [30]. In addition, drunk driving is a major risk factor; speeding and drunk driving have always been the focus of attention in the prevention of traffic collisions. Our research could not assess whether the impact of speeding and drunk driving on sanitation worker road traffic collisions is the same as on road traffic collisions for other groups of people, but it is urgent for the urban traffic management department to strengthen monitoring and intervention in driver’s speeding and drunk driving.

It is also worth noting that many collisions are caused by sanitation workers themselves, mostly by crossing the road at will. This behavior may be a result of a lax public attitude towards litter, the absence of legal control on environmental protection, the excessive pursuit of efficiency in urban governance, and the lack of basic education and vocational training plus risk-taking behavior in sanitation workers themselves.

Public tolerance of litter and the absence of legal controls for environmental protection result in road garbage. The Chinese have a poor awareness of environmental protection, and the overall sense of environmental responsibility is very low [13, 31]. Much road garbage in China comes from littering and debris thrown during garbage removal; 90% of road garbage comes from litter thrown from car windows [14]. Therefore, sanitation workers must repeatedly walk on the road to deal with garbage. In China, the suppression of littering behavior is mainly based on the self-discipline of citizens and lacks mandatory measures or effective consequences. Chinese laws and regulations on environmental protection all deem littering a crime, but compared with the United States, Singapore, Japan, Italy, and other countries, punitive measures appear more tolerant. At the same time, China does not usually supervise such behavior, which makes it difficult to implement effective consequences. As a demonstration, the “garbage classification” regulation carried out in Shanghai corrected, in just 2 months, residents’ unreasonable garbage disposal behavior that had been going on for years [32]. The program was executed by imposing high fines and strong supervision and enforcement. Texas has eliminated local residents’ littering on the highways through public advocacy centered on “Don’t discredit Texas” [33]. It is suggested that linking the “window littering” with “killing sanitation workers” in public advocacy may help dispel citizens’ uncivil behavior.

The excessive pursuit of efficiency in urban governance has fueled the emergence of dangerous behaviors in sanitation workers. Given the abundant and cheap labor force in developing countries, the decision of sanitation departments or urban managers not to mechanize cleaning only considers economic benefits and ignores the safety of sanitation workers. In addition, strict management of sanitation workers also reflects the excessive pursuit of efficiency. From the end of 2016, the “cigarette butt revolution” initiated by the Xi’an municipal government of Shaanxi Province is a typical example: there, in the interest of city beautification, every cigarette butt found on the road incurred a fine of one yuan against sanitation workers. Vehicles and pedestrians make it difficult for sanitation workers to ensure that the roads were consistently kept clean. Thus, sanitation workers are compelled to cross the road frequently to pick up garbage to avoid incurring fines. Although efficiency is an important value in urban governance, efficiency at the expense of fairness is the exchange of sanitation workers’ lives for urban cleanliness.

The lack of basic education and vocational training for sanitation workers limits their perception of behavioral consequences and choice of behavior. The overall level of education of sanitation workers is low, most having attended only primary school, if any [11, 24]. Agbola and Gutberlet determined that the level of perception of occupational health risk of waste handlers may be affected by their level of education [12]. China’s sanitation workers do not have qualification restrictions for occupational access, and there is no education or training for sanitation workers, pre-service or in-service. It is possible that their violation of traffic rules and operating regulations were also related to the lack of vocational education and training.

Objective factors such as bad weather and dim light also contribute to sanitation worker road traffic collisions [34]. It is difficult to predict and control the occurrence of objective factors; all one can do is to take reasonable protective measures. For example, the Chengdu Government of Sichuan Province has issued the “Severe Weather Sanitation Work Plan” to cope with the adverse effects of weather on sanitation workers. Dim light can be mitigated by increasing the density, location, and start-up time of lighting equipment such as street lamps, and tunnel areas need to be specially considered in the lighting safety design [35, 36]. Even the functioning of road warning signs and traffic lights should be taken seriously in urban infrastructure management. For sanitation workers, fully equipping them with reflector jackets and luminous helmets (or headlamps) can increase their visibility under such circumstances.

Some of the variables included in the analysis of this research had low media coverage rates, such as sex of victims, causes of collisions, and sources of victim compensation. We have no significant evidence that this is associated with news source or region. However, victim compensation may remain an ignored area in the media and policy, which may encourage employers to violate workers right and compensations in case of occupational diseases or injuries. In addition, the media’s underreporting of certain important information indicates the necessity of developing a surveillance system for such incidents.

China’s aging sanitation workers have low incomes, low social status, and low education, and they experience excessive road traffic collisions. They are a vulnerable urban group with relatively limited social resources and a relatively high risk of death [37]. In order to reduce the occurrence of road traffic collisions in sanitation workers and ensure equal opportunities for health, measures that specifically target these socially disadvantaged groups should be implemented.

### Limitations

There are some limitations to our research. First, the information that has been analyzed in this study of factors contributing to sanitation worker road traffic collisions has been limited to online news sources, which has restricted our understanding of the issue. Mass media tend to report only serious collisions to draw attention to their stories. However, considering the difficulty of obtaining public data in China, using online news as a data source also provides a way to study major social public health issues. Second, web search engines may not cover all collision news, leading to the omission of certain collisions from our data. Third, affected by the inconsistency of the content structure of collision news, there was a lot of missing data in the encoding process of some variables, which may lead to certain bias in the analysis and discussion. Fourth, in the analysis of causes of collisions, this study compared only the frequency of occurrence of different factors. The specific contribution of different factors and the collinearity between them need to be studied further. Fifth, the current research on sanitation worker road traffic collisions is limited, so it is difficult to compare and discuss the results of this study. Sixth, the effect of protective devices for sanitation workers such as reflective clothing and warning signs is worthy of further exploration in follow-up research, including mechanism of effect, degree of effect, and style design. Finally, considering the source of the data in this study, this article only conducted a descriptive analysis, but did not conduct an analysis of the correlation between factors. In the future, it may be necessary to conduct a more specific sample survey on the occupational groups to analyze the occurrence of road traffic collisions in depth.

## Conclusion

This study is the first analysis of road traffic collisions involving China’s sanitation workers, focusing on occurrence time and location, characteristics of victims, and causes of collisions. As urbanization progresses, sanitation workers have become an occupational group vulnerable to road traffic injuries. There is a need to raise awareness about occupational protections for this vulnerable group in developing countries.

## Data Availability

The database used in the current study was collected from online news by researchers through search engines. The database generated during the current study is not publicly available due to privacy restrictions but are available from the corresponding author on reasonable request.
